# A New Artificial Urine Protocol to Better Imitate Human Urine

**DOI:** 10.1038/s41598-019-56693-4

**Published:** 2019-12-27

**Authors:** Neslihan Sarigul, Filiz Korkmaz, İlhan Kurultak

**Affiliations:** 1 Institute of Nuclear Science, Hacettepe University, 06532 Ankara, Turkey; 2 Atilim University, Faculty of Engineering, Biophysics Laboratory, 06836 Ankara, Turkey; 3 Trakya University, Faculty of Medicine, Department of Nephrology, 22000 Edirne, Turkey

**Keywords:** Molecular biophysics, Diagnostic markers

## Abstract

Artificial urine has many advantages over human urine for research and educational purposes. By closely mimicking healthy individuals’ urine, it may also be important in discovering novel biomarkers. However, up until now, there has not been any specific protocol to prove the similarity in terms of the chemical composition at the molecular level. In this study, a new artificial urine protocol is established to mimics the urine of healthy individuals. The multi-purpose artificial urine (MP-AU) presented here is compared with two other protocols most cited in literature. Furthermore, these three protocols are also compared with samples from 28 healthy young individuals. To do so, attenuated total reflection-Fourier transform infrared spectroscopy (ATR-FTIR) is used, according to which MP-AU shows a significantly close similarity with human urine. In formulating MP-AU, the infrared spectra of nine compounds is provided, making possible the band assignment of some absorption bands to certain compounds. Given its properties, the MP-AU protocol introduced here is both economical and practical, making it useful when designing comparative-controlled experiments.

## Introduction

Urine is a rich bodily fluid in terms of its contents. Over 3000 metabolites have been defined in urine in the past three decades^1^. The components of urine have the potential to serve as important biomarkers and diagnose numerous diseases. However, studying with urine has challenges. Determining the exact composition of urine is both difficult and expensive. The composition changes according to gender, age, race, food intake, presence of medication and exercise^2–4^. Furthermore, it changes throughout the day in the same individual according to the requirements for normal bodily functions. Another difficulty is the collection and storage problems, which have been emphasised in many studies^5,6^. Possible existence of communicable pathogen agents in urine specimens is also a risk factor that a researcher should take into account. Due to these downsides, the demand for artificial urine (AU) for research purposes is increasing as it is both practical and fast to obtain. The formulation can be easily manipulated to design comparative-controlled experiments.

AU specimens have been studied in many different research areas such as dermatology, urology, nephrology, pharmacology^7–9^. There are numerous protocols for preparing AU in literature. These formulas are different in composition, quantity, concentration and method of preparation, and thus they serve different purposes. While a certain formula is better suited for studying the growth of urinary pathogens^10^, another formula is better for studying components of urine for different pathological conditions^11^, or kidney and urinary tract cells *in vitro*^12^. When these protocols are investigated, important urine components are seen to be missing such as urea^8,13–17^, creatinine or uric acid^7,8^. Such protocols cannot be used as representative of human urine.

Until now, the closest AU formulation to urine has been proposed and published by Chutipongtanate and Thongboonkerd^12^. The reference values of urine components in this protocol were determined from the studies by Taylor and Curhan^3^, and Cameron *et al*.^18^ together with the references listed in Medline Plus at the time of the study in 2010. Taylor and Curhan (2007) published their reference values based on post-menopausal women above 65 years of age. On the other hand, the mean age of the control group in the study by Cameron *et al*. is 49 and the evaluation of their health conditions is undefined. Thus, although the methodology of Chutipongtanate and Thongboonkerd is scientifically accurate, the urine composition depends highly on the age and hormonal status along with various other factors. Another AU formulation belongs to Brook and Keevil^10^, which uses the study by Altman^19^ in designing the list of components and their normal ranges. The formulation includes bicarbonate although it is not naturally found in human urine unless there is a pathological condition that alkalizes the urine^20^. In the literature, there is no confirmation about whether or not these AUs correlate with the urine of healthy individuals. Therefore, how they will be used in human clinical studies is still ambiguous. A reliable AU formula that is the closest match to healthy human urine is a necessity.

Technological developments in analysis techniques like MS, MS-TOF, NMR, chromatography (GC-MS, LC-MS/MS) paved the way to identifying over 3000 constituents in urine^1^. However, it is neither practical nor applicable to individually determine all of ingredients for every patient. The method to be used for urine analysis should yield results to include the effects of almost all molecules in urine in one output. In this respect, Fourier Transform Infrared (FTIR) spectroscopy is a suitable method for such analysis. It provides information on the chemical composition of samples. There is a rapidly increasing literature on the applications of infrared spectroscopy for the early detection of diseases such as cancer by analysing biofluids, tissues or cells^21–29^. FTIR has a great potential in clinical laboratory due to its ease of use, affordable cost, transportability and its sensitivity.

The main aim of this study is to formulate a new AU, composed of adequate number of components within the physiological ranges of healthy human urine. The proposed formulation is compared to two other AUs published in literature^10,12^. Here, the degree of resemblance between human urine and AU is determined at the molecular level by using attenuated total reflection-Fourier transform infrared (ATR-FTIR) spectroscopy. In this study, we have collected and scanned human urine from 28 individuals to compare the infrared spectrum of the average morning urine to that of our multi-purpose artificial urine (MP-AU). To the best of our knowledge, this is the first study showing infrared spectra of the first morning urine of 8 hour-fasting healthy individuals. We have also obtained spectra of the constituents of MP-AU, so that it was possible to identify the characteristic peaks of important urine components such as urea, uric acid and creatinine.

## Methods

### Sample preparation

Human urine is composed primarily of water (95%). The rest is urea (2%), creatinine (0.1%), uric acid (0.03%), chloride, sodium, potassium, sulphate, ammonium, phosphate and other ions and molecules in lesser amounts^30^ (Table 1). Protein is only found in trace amounts compared to their values in blood plasma. A recent study published by Bouatra and co-workers (2013)^1^ revealed more than 3000 components in human urine. From the complete list of components published in the same study, there are over 90 compounds with 100% occurrence composing the urine regardless of the gender or the time of the day that the urine is collected. For a practical and economical AU formulation, the number of ingredients to be used in MP-AU is kept to a minimum; thus, only the components with relatively higher concentration compared to others are selected and used.

**Table 1:** Physiological ranges of selected compounds in healthy human urine.

Property and Composition	Molar Mass (g/mol)	Normal Range in humans (reference age in years)	Molarity (mmol/1.5 L)
Volume		0.8–2 L	
pH		4.5–8.0	
Specific gravity (SG)		1.002–1.030 g/ml (all)	
Osmolality		150–1150 mOsm/kg (>1)	
Urea (CH_4_N_2_O)	60.06	10–35 g/d (all)	249.750
Uric Acid (C_5_H_4_N_4_O_3_)	168.11	<750 mg/d (>16)	1.487
Creatinine (C_4_H_7_N_3_O)	113.12	Males: 955–2936 mg/d	7.791
		Females: 601–1689 mg/d (18–83)	
Citrate (C_6_H_5_O_7_^3−^)	192.12	221–1191 mg/d (20–40)	2.450
Sodium (Na^+^)	22.99	41–227 mmol/d (all)	92.625
Potassium (K^+^)	39.10	17–77 mmol/d (all)	31.333
Ammonium (NH_4_^+^)	18.05	15–56 mmol/d (18–77)	23.667
Calcium (Ca^2+^)	40.08	Males:<250 mg/d	1.663
		Females:<200 mg/d (18–77)	
Magnesium (Mg^2+^)	24.31	51–269 mg/d (18–83)	4.389
Chloride (Cl^−^)	35.45	40–224 mmol/d (all)	88.000
Oxalate (C_2_O_4_^2−^)	88.02	0.11–0.46 mmol/d (all)	0.277
Sulphate (SO_4_^2−^)	96.06	7–47 mmol/d (all)	18.000
Phosphate (PO_4_^2−^)	94.97	20–50 mmol/d (>18)	23.33

Table 1 shows the physiological ranges of selected compounds in healthy human urine. The data has been retrieved from Mayo Medical Laboratories^31^, which is a global reference laboratory operating within the Mayo Clinic’s Department of Laboratory Medicine and Pathology. The mean values of the ranges given for each component are used to prepare MP-AU. The concept of ‘normal’ also depends on gender for calcium and creatinine. For these two components, a normal range is determined for both male and female that falls within the normal for both sexes in this study. For example, a range of 955–1689 mg/d of creatinine is taken to be the normal physiological range, so that, even at extreme points, the given amount is still ‘normal’ for both genders.

The normal volume range of urine output is 0.8 to 2 l/d for a person having a normal fluid intake. For molarity calculation of each compound, 1.5 l/d is assumed for an average person^32–34^. The right-most column in Table 1 shows the basis of MP-AU composition as well as the concentrations of each compound. Table 2 provides a complete list of the MP-AU components and their final concentrations. All chemicals used in this study are in powder form, purchased from Merck (Germany) and used without further purification. The components are dissolved in 100 ml double-distilled water using a magnetic stirrer (Heidolph, Germany) rotating at 250–500 rpm. During mixing, the temperature of the solution is kept constant at 37.5 °C using the heater function of the stirrer. The same procedure is followed for all AUs.

**Table 2:** The composition of MP-AU.

	Molarity (mM)	Quantity (g/100 ml)
Na_2_SO_4_	11.965	0.1700
C_5_H_4_N_4_O_3_	1.487	0.0250
Na_3_C_6_H_5_O_7_.2H_2_O	2.450	0.0720
C_4_H_7_N_3_O	7.791	0.0881
CH_4_N_2_O	249.750	1.5000
KCl	30.953	0.2308
NaCl	30.053	0.1756
CaCl_2_	1.663	0.0185
NH_4_Cl	23.667	0.1266
K_2_C_2_O_4_.H_2_O	0.19	0.0035
MgSO_4_.7H_2_O	4.389	0.1082
NaH_2_PO_4_.2H_2_O	18.667	0.2912
Na_2_HPO_4_.2H_2_O	4.667	0.0831

After the preparation of AUs, they are tested using semi-quantitative urine dipsticks (Mission Acon, San Diego, USA). The tests are performed according to the manufacturer’s instructions. Also, a Mettler-Toledo pH meter (Schwerzenbach, Switzerland) is used for pH measurements. A freshly prepared solution is always used for measurements. When the components are added in the order provided in Table 2, the pH of the solution is stabilized around 6.00 ± 0.08 after 24 h at 37 °C. Therefore, for pH sensitive studies, it is recommended to prepare the AU solution one day prior to the research study. The repeatability of the MP-AU is tested by preparing three samples and recording their FTIR spectra (Supplementary Fig. S1). Results show that the MP-AU solution is reproducible.

In this study, we prepared three artificial urine batches. Together with our own formulation (MP_AU), other two are based on the protocol published by Chutipongtanate and Thongboonkerd (called CT-AU) (2010)^12^, and Brooks and Keevil (called BK-AU) (1997)^10^. The list of components and concentrations can be found in Supplementary Table S1. In the preparation of BK-AU, yeast and peptone are not used. In the original study, these compounds were used to examine the growth of urinary pathogens^10^. The measurements are also repeated three times for CT- and BK-AU (Supplementary Fig. S1). Average of three measurements for all AU formulations are calculated and used for comparison with human urine.

The infrared absorbance of each component in the formulation of MP-AU is obtained by dissolving the appropriate amount (as in Table 2) in 100 ml double-distilled water. In this way, 13 batches of solutions, each containing a different component are prepared and measured individually. As a result, a spectral library of each component used in this study is obtained.

### Urine collections from volunteers

This study was performed in accordance with the relevant guidelines and regulations for studies in human subjects and was approved by the Atilim University Human Studies Ethics Committee (Ref.Number: 59394181-604.01.01-5229). All volunteers signed an informed consent form. Twenty-eight individuals in the age range 20–40 volunteered for the study, ten female and eighteen male. First, the volunteers were interviewed to determine their general health statues. Excluded from the study were those stating complaints related to urinary system, as well as those using medications and finally, those with a history of acute or chronic disease. The remaining volunteers were given questionnaire related to their daily habits, addictions and medications. All participants in this study were determined as healthy based on the questionnaire, standard medical evaluations, and dipstick test applied to their urine samples. The urine samples were collected at the Health Centre of Atilim University. In this study, first morning urine samples from 8h-fasting volunteers were preferred over spot urine samples considering reliability^35^. Samples were collected in sterilized, individually packaged cups. The dipstick tests were applied to freshly-collected mid-stream urine. The fasting blood glucose, blood pressure, blood saturated O_2_, heart beat rate and body temperature were measured and recorded. The urine samples were kept in a refrigerator (4 °C) and measured with ATR-FTIR within the next two hours after collection. The clinical datasets obtained from volunteers in this study are available from the corresponding author on reasonable request.

### ATR-FTIR

The infrared absorbance spectrum is obtained with a Nicolet 6700 (Thermo Scientific, USA) spectrometer equipped with a diamond Attenuated Total Refraction (ATR, ConcentratIR2, Harrick, USA) accessory with 10 internal reflections. A DTGS (Deuterated Tri Glycine Sulphate) detector is used for each sample measurement, where a background interferogram is recorded with a clean diamond surface. Then, 5 µl of sample is pipetted onto the diamond and dried under a gentle stream of nitrogen for 15 minutes. Since the absorption of water (H-O-H stretching) interferes with urea absorption, drying the sample was preferred prior to data acquisition. The drying time is optimised with a sample urine dried for 25 minutes, during which, the sample is scanned every 5 minutes. It was observed that the O-H stretching vibration at ~3400 cm^−1^ disappears, and that region remains constant in amplitude after 15 minutes. This way, only the excess water is removed^36^. All samples are measured by collecting and averaging 120 scans for a final resolution of 4 cm^−1^. The spacing between two real points is 0.96 cm^−1^. The spectra are recorded without any digital signal enhancement methods. The sample chamber of the interferometer is continuously purged with N_2_ during measurements to eliminate the atmospheric variations in water vapour. The second derivative of spectra is calculated using the Savitsky-Golay method with 17 points of smoothing. Spectra collection and derivative calculations are performed with the spectrometer software OMNIC version 8.2.388 (Thermo Scientific, USA).

## Results

**Figure 1:**
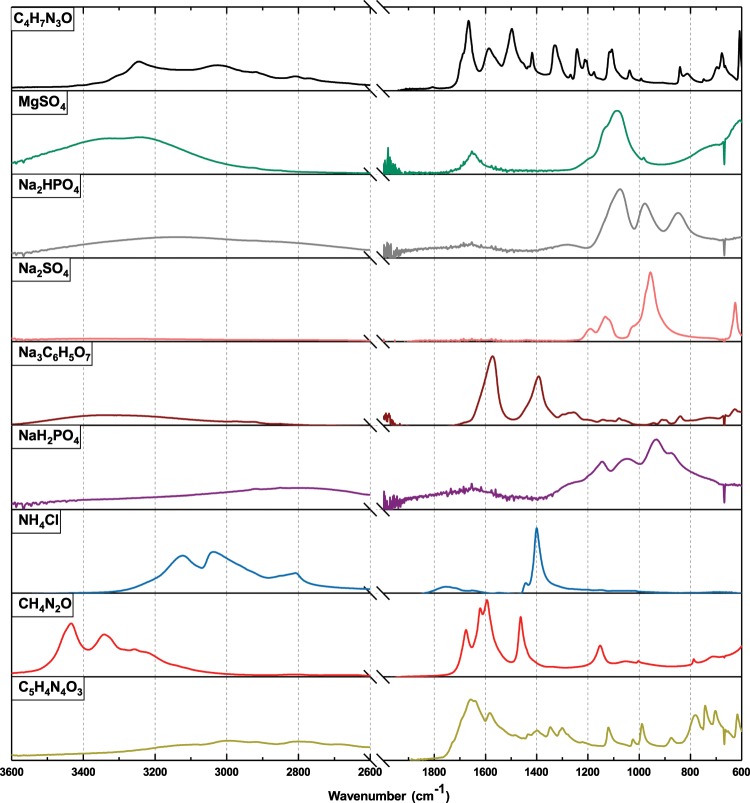
Infrared absorbance spectra of nine compounds used in the MP-AU formulation. Scaling of the vertical axes are not equal. Relative absorbances are given in 3D plot in Supplementary Fig. S2. The signal-to-noise ratio is limited in ATR diamond in 2600–2000 cm^−1^ region, and thus is excluded from the display. NaCl, KCl and CaCl_2_ did not have any absorption in the measured window and thus, are excluded from both figures. Also the absorption of K_2_C_2_O_4_ is very weak, and thus its spectrum is excluded from the figure. Peak positions are presented in Supplementary Table S2.

In practice, finding a true spectral match between any AU and human urine is impossible, but the degree of resemblance is a reliable measure. Three AU batches are compared in terms of SG, pH and IR spectrum as the measurement of match to human urine. A statistical analysis is also applied on IR spectra. After water, the main component of urine is urea. In fact, the spectral contribution of urea to AU is dominant compared to other components (Fig. 1 and Supplementary Fig. S2). Creatinine and sodium citrate are the second and third components with profound spectral contributions.

BK-AU, CT-AU and MP-AU are prepared as described in Method. BK-AU and CT-AU share common chemicals in their protocols. However, while both includes bicarbonate, this compound is not naturally found in healthy human urine^1^. Therefore, bicarbonate was not used in the MP-AU formulation. The concentrations of chemicals are different when BK-AU and CT-AU are compared since the two studies rely on different literature regarding urine composition. Despite the differences in the formulas, all AUs have acceptable physical properties in terms of SG and pH (Table 3), and all ingredients are within the physiological ranges based on the Mayo Clinic test catalogue. On the other hand, urine is a complex solution by nature and comparing any AU formulation with human urine based on just two parameters (SG and pH) is a blind approach at best. Therefore, we utilized a spectroscopic analysis using FTIR to point out similarities and/or differences among AUs, and compared them with human urine samples.

**Table 3:** Physical properties of all AUs. Please refer to the text for abbreviations used in the table.

	CT-AU	BK-AU	MP-AU
pH	6–7	7–8	5.5–6.5
SG	1.010–1.015	1.005–1.010	1.010–1.020

### Human urine

**Figure 2:**
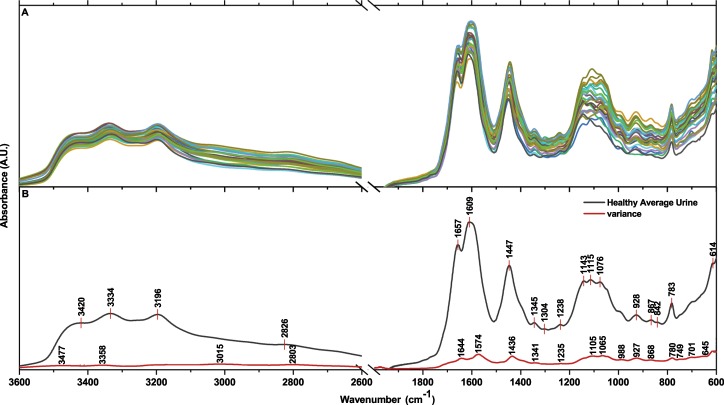
Infrared absorbance spectra of 28 healthy individuals aged 20–40 years. (**B**) Calculated average spectrum of human urine is shown in black and the variance among 28 urine spectra is shown in red.

The absorbance spectra of urine samples from 28 healthy individuals are shown in Fig. 2. Generally, the spectra of human urine show the same profile, particularly in the 1800–1200 cm^−1^ window of the spectrum. However, there are significant variations among the individuals in the 1200–800 cm^−1^ range. It can be due to diet, gender and metabolism differences. When the variance is calculated, the most prominent peaks are seen to be at 1644, 1574, 1436, 1105 and 1065 cm^−1^. According to our database of spectra obtained from individual ingredients (Fig. 1), variations in the level of creatinine, citrate, urea, phosphate and uric acid may have contributed to these differences along with other urine components. The average of 28 urine samples is calculated and used hereafter for comparison (Fig. 2).

### Artificial urines

**Figure 3:**
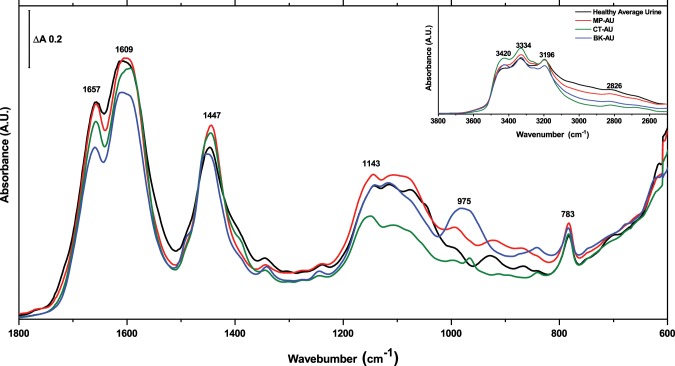
Infrared absorbance spectrum of MP-AU (red), CT-AU (green), BK-AU (blue) together with the spectrum of the average healthy urine from 28 participants (black) in fingerprint region. Inlet figure shows the same spectra in the 3800–2500 cm^−1^ region.

The calculated average human urine is compared with the three AU spectra as shown in Fig. 3. The band profiles and positions are seen to be similar at many points, namely 1657, 1609, 1447, 1143 and 783 cm^−1^ (Fig. 3). These bands mainly originate from urea. However, there are also significant variations and deviations from these positions as discussed in detail in what follows.

All spectra share common features in the higher frequency region (4000–2500 cm^−1^) (Fig. 3-inlet). The two highest absorbances centred around 1600 cm^−1^ and 1445 cm^−1^ are mainly due to urea with relatively minor contribution from creatinine. The slight differences in the absorbance of AUs are mainly due to the differences in urea concentration in respective formulations. On the other hand, the region between 1200 cm^−1^ and 800 cm^−1^ shows significant variations. The most prominent difference is the absorbance at 975 cm^−1^ in BK-AU (Fig. 3). At this position, all AU spectra have different number of peaks at different positions. This region is the collection of a number of peaks originating mainly from urea, uric acid, creatinine and sodium phosphate (Fig. 1 and Supplementary Fig. S2). Therefore, the slight variations in the concentration of these compounds yield a very different profile in the spectrum.

**Figure 4:**
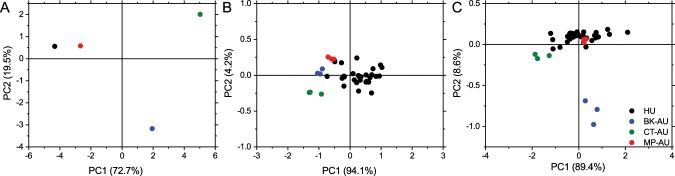
Score plot of principal component analysis applied to average spectrum of human urine (HU) and artificial urine measurements. Plot compares spectra in full wavenumber range (4000–600 cm^−1^) (**A**). Score plot of all IR measurements compared in 1400 cm^−1^ range (**B**) and in 1000–800 cm^−1^ range (**C**).

Principal component analysis (PCA) is used for the differentiation of three average artificial urine spectra with respect to average human urine in the whole wavenumber region (4000–600 cm^−1^) (Fig. 4A). The first principal component (PC1) (72.7%) and the second principal component (PC2) (19.5%) account for 92% of total variance. The score plot shows that MP-AU shows greater similarity to average human urine compared to other AU formulations when the whole spectra are taken into consideration. This analysis is also applied to all three measurements for AUs and to all human urine measurements of 28 healthy individuals. Comparisons are shown in two regions of the IR spectrum, i.e., 1400–1200 cm^−1^ (Fig. 4B) and 1000–800 cm^−1^ (Fig. 4C). In both regions, MP-AU is more successful in imitating spectral features of human urine. While BK-AU is more successful compared with CT-AU in the 1400–1200 cm^−1^ region, it is the opposite in the 1000–800 cm^−1^ region. Which bands or compounds account for these differences are discussed in the following sections.

### Human urine and MP-AU comparison

**Figure 5:**
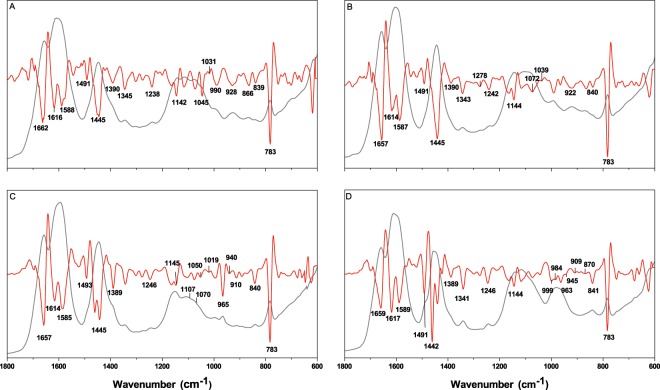
Infrared absorbance spectrum of the average human urine (**A**), MP-AU (**B**), CT-AU (**C**), BK-AU (**D**) shown in grey and their second derivative profiles shown in red.

There is a satisfactory match between the spectrum of MP-AU and human urine when absorption (grey trace) and second derivative profiles (red trace) are compared (Fig. 5A,B). Two spectra are not significantly different at the 0.05 level based on Mann-Whitney test (Z = −1) (Supplementary Table S3). The region between the 1800–1400 cm^−1^ is nearly common as suggested by second derivative profiles. However, the peak at 1390 cm^−1^ is different. The absorption (grey trace) at this point in MP-AU is not as high as in urine. This absorption is due to the presence of sodium citrate (Fig. 1 and Supplementary Table S2). Although the amount of citrate is the mean value of the physiological range, the mismatch may indicate a vast amount of citrate in volunteers. Citrate concentration in urine can vary depending on diet. For example, in DASH (Dietary Approaches to Stop Hypertension)-style diet (rich in vegetables, fruits, whole-grains, low-lipid foods, fish, meat, nuts and beans; limited in red-meat, sugar-sweetened fruits, beverages and fats) the amount of citrate increases in the urine^37^.

The peaks at 1343, 1301, 1278 and 1242 cm^−1^ are nearly the same in position (Fig. 5B-grey trace) and in terms of the relative ratio of amplitudes, except that the peak at 1301 cm^−1^ is less obvious from the absorbance spectrum. MP-AU provides the best match in this 1350–1200 cm^−1^ region when compared with other AUs. In the 1200–1000 cm^−1^ region, instead of the 1031 and 1045 cm^−1^ peak in the human urine (Fig. 5A-red trace), there is one peak at 1039 cm^−1^ in MP-AU (Fig. 5B-red trace). The spectral match between the two spectra in the region 1000–600 cm^−1^ is satisfactory. One difference is at position 928 cm^−1^ in the urine absorption spectrum. This band is located at 922 cm^−1^ in MP-AU. When second derivative profiles are examined, this peak is seen to be the superposition of two peaks. The major contributor is a peak at 929 cm^−1^, with minor contribution from another peak at 916 cm^−1^ in the urine spectrum. However, in MP-AU the amplitude of 929 cm^−1^ absorption is lower, which shifts the position of the envelope down to 922 cm^−1^. This could be due to the rich content of human urine providing additional absorbers at 929 cm^−1^ that are not included in MP-AU.

### Human urine and CT-AU comparison

The 1800–1400 cm^−1^ region of the absorbance spectrum is very similar to the spectrum of urine in the same region, which is mainly correlated with the urea content (Fig. 5A,C). The CT-AU spectrum is also successful in matching the shoulder at 1390 cm^−1^. The normal physiological range for citrate is 0.2–1.2 g/d^31^. In CT-AU formulation, the amount of citrate corresponds to 2 g/d (assuming 1.5 L urination per day), which is more than the maximum point of the physiological range. It appears that the average urine spectrum reflects a vast amount of citrate as indicated in the previous section. When the entire fingerprint region of CT-AU spectrum is compared with human urine spectrum using Mann-Whitney test, at the 0.05 level, two spectra are significantly different (Z = −13.5) (Supplementary Table S4).

In the human urine spectrum, the band at 1238 cm^−1^ is seen to be located at 1246 cm^−1^ in the CT-AU spectrum (Fig. 5C-red trace). What remains unclear, however, is whether the two bands reflect the vibration of the same molecular group, or they are unrelated absorptions. Human urine has multiple absorbances in the 1200–1000 cm^−1^ region. Although most of the components are common between urine and CT-AU, there are significant differences in terms of the relative ratios of the bands and, thus, the overall profile is different. Peak positions are generally similar, except the one at 1045 cm^−1^ in urine spectrum, which is located at 1050 cm^−1^ in CT-AU. Also, the peak at 1031 cm^−1^ is missing in the CT-AU spectrum. The most prominent amplitude difference is at 1107 and ~1070 cm^−1^. An insufficient amount of phosphate compounds, creatinine and uric acid could have contributed to the difference at these positions. The region is a superposition of many vibrational groups found in various molecular formations such as lipids, proteins, glucose and its derivatives, and in nucleic acids. Therefore, matching an AU spectrum with that of human urine is nearly impossible in this 1200–1000 cm^−1^ region. Another important difference is the peak at 965 cm^−1^ seen in CT-AU, but not in human urine spectrum. We do not have enough data to discuss the possible cause of this band since none of the urine components we measured absorb at this position. The last point in the comparison of CT-AU with human urine is the absence of 928 and 866 cm^−1^ peaks in CT-AU.

### Human urine and BK-AU comparison

The general spectral profile of BK-AU is also similar to human urine at many points (Fig. 3). The three highest absorbances at positions 1657, 1609 and 1447 cm^−1^ in human urine spectrum match in terms of general appearance, but slightly vary in peak position in the spectrum of BK-AU (Fig. 5A,D-grey traces). However, the peak at 1491 cm^−1^ (seen as a shoulder on the 1446 cm^−1^-peak in the absorbance spectrum) is more pronounced in BK-AU. This position might be attributed to creatinine (Fig. 1 and Supplementary Table S2). The amount of creatinine used in BK-AU formulation is within the normal physiological range although it is less than the mean value. In fact, the amount of creatinine in MP-AU formulation is more, but the absorbance at ~1490 cm^−1^ is less. Therefore, such a pronounced peak cannot be explained by the amount of creatinine. On the other hand, we observed a rise of amplitude at this position when sodium sulphate is added to the solution while preparing MP-AU, although sodium sulphate does not have an absorbance in this region. Magnesium sulphate does not create this effect. Based on this experience, we believe that the increased absorbance at ~1490 cm^−1^ is related with the amount of sodium in the formulation of BK-AU. The total sodium concentration used in BK-AU formulation is beyond the physiological range and is the maximum among the three AUs. A detailed comparison of chemical contents is presented in the next section.

A mismatch of spectral profiles between BK-AU and urine is seen at ~1390 cm^−1^ (Fig. 3). The amplitude at this position is more in the urine spectrum. This peak was attributed to the citrate in previous sections. However, many C-H vibrational modes also absorb in this region such as the symmetric deformation mode of methyl group at 1380–1390 cm^−1^^38,39^. In human urine, there are a number of CH_3_ sources together with creatinine^1^, such as 3-Methylhistidine, acetic acid, acetone, Alpha-Hydroxyisobutyric acid, 5-Methyl-2-hexanone, etc. that contribute to the absorption at 1390 cm^−1^. The lack of such compounds could have resulted in the lower amplitude at 1390 cm^−1^.

The 1345 cm^−1^ peak in the human urine spectrum is located at 1341 cm^−1^ in BK-AU spectrum (Fig. 5A,D-red traces). This band originates mainly from urea (1331 cm^−1^), uric acid (1346 cm^−1^), and creatinine (1333 cm^−1^), and receives minor contribution from sodium citrate (Supplementary Table S2). In the formulation of BK-AU, the concentration of urea is within the first 20% portion of the normal physiological range, which is quite less than the mean value. The concentrations of uric acid and creatinine are also less than average i.e., 13.5% and 33.5% portion, respectively. The difference at the position of the band is due to the different ratios of these components relative to one another, so that the superposed band is slightly shifted in location. Another difference in the spectrum of BK-AU is the peak located at 1246 cm^−1^ (Fig. 5D). In human urine spectrum this peak is located at 1238 cm^−1^. Unfortunately, it is not clear whether the two bands are due to the same molecular group and there is a shift in position, or the two bands originate from different molecular groups. The same peak is also observed in CT-AU, as pointed out earlier. Additionally, the problem in the 1200–1000 cm^−1^ region seen in CT-AU is also observed in BK-AU. As mentioned earlier, a perfect match in this window of the spectrum is nearly impossible due to the number of ingredients necessary for an economical AU.

The biggest difference in the spectrum of BK-AU is the relatively excessive absorption centred at ~975 cm^−1^. This band is a superposition of five different components at 999, 984, 963, 945 and 909 cm^−1^ as revealed by the second derivative profile of the spectrum. A similar peak is also observed in CT-AU at 965 cm^−1^, but the band amplitude is much smaller than the one in the BK-AU spectrum. In the range between 940–1020 cm^−1^, bicarbonate, di-sodium hydrogen phosphate and urea have absorptions (Fig. 1 and Supplementary Fig. S3). When the individual absorption spectra of these three compounds are added (spectral addition), the resulting amplitude in the corresponding region is much less than what is observed in BK-AU. This proves the formation of a compound among the constituents.

The peak at 866 cm^−1^ in the urine spectrum is missing in BK-AU, as was the case in CT-AU. The last difference between the two spectra is at 841 cm^−1^ in the BK-AU spectrum. This peak is more pronounced in BK-AU spectrum relative to the CT-AU and human urine spectra. It is mainly due to the creatinine (842 cm^−1^). Magnesium sulphate also contributes with a baseline, giving the band its final position and profile as far as we observed during our experiments. In BK- and CT-AU, bicarbonate also absorbs at 832 cm^−1^ that contributes to the amplitude but does not change the position of the 841 cm^−1^ peak.

In the whole fingerprint region, BK-AU and human urine spectra are significantly different at the 0.05 level based on Mann-Whitney test (Z = −10.8) (Supplementary Table S5).

### Comparison of artificial urines in terms of chemical content

**Figure 6:**
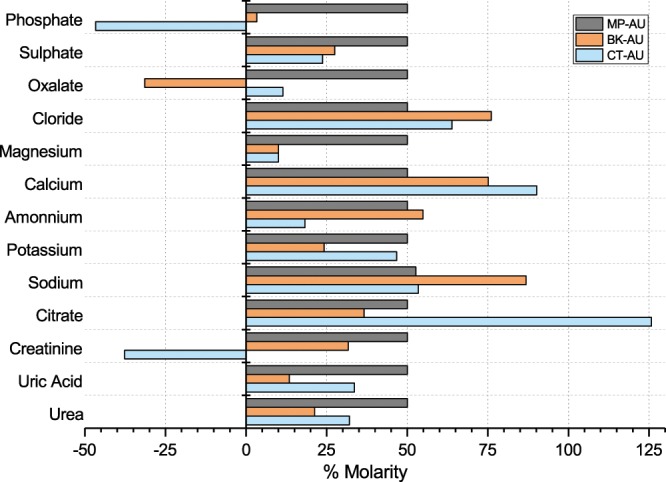
Relative amount of common compounds used in all AUs. Horizontal axis represents the molarity percentage of the normal physiological range for each compound. All normal range values are normalized so that the minimum point of the normal range is set to 0% and the maximum point of the normal range corresponds to 100%.

The list of chemicals used in all three AUs are mainly the same; however, concentration of each component is different (Supplementary Table S1). Figure 6 shows the amount of each component relative to its normal physiological range^31^. MP-AU comprises of thirteen components, each at the level of 50%, which is the mean of the normal physiological range. A negative value in the figure represents insufficient amount of the corresponding chemical. The chemical composition of CT-AU formulation has deficiencies. To begin with, the amount of citrate exceeds the normal range (~125%). In the same vein, the amount of calcium and chloride are more than the mean values, 90% and 64%, respectively. While creatinine and phosphate are insufficient, the amount of urea, uric acid, ammonium, magnesium and sulphate are close to the minimum values of normal ranges. In the formulation of BK-AU, the amount of sodium, calcium and chloride are more than the mean values, 87%, 75% and 76%, respectively. On the other hand, urea, uric acid, potassium, magnesium, sulphate and phosphate are quite less than the mean of the normal physiological range. Although, these compounds are still within the normal range for a healthy human, the amount of oxalate is insufficient.

## Discussion

Human urine studies in the literature use mainly two different methods of urine collection: spot urine and 24 h urine^40^. The dependence of urine composition on diet, gender, age and race is already well-known. Furthermore, this composition of urine shows differences throughout the day for the same person^41^. Therefore, comparing spot urine samples among individuals does not give reliable results. On the other hand, 24 h urine collection is difficult in itself for the participant since he/she should carry the collecting container throughout the day and keep it cool at the same time. Another challenge is contamination, to prevent which, a preservative chemical is used in the collecting container, thus compromising the chemical composition for spectral analysis.

In this paper, the first morning urine was collected from participants who were fasting for at least 8 h. As such, the composition is close to the 24 h-collected sample without the risk of contamination^35,41–44^. Fasting is a necessary step so that the collected urine depends more on the metabolism and less on the food consumed at dinner.

For this study, urine samples were obtained from 28 individuals at 20–40 age range. The individuals were examined clinically by a medical doctor and whose urine samples were analysed with dipstick test. Our first observation is that, although urine samples are different among individuals, the FTIR spectra are mostly similar. Another study performing FTIR analysis on rat urine shows that the urine of 34 normal rats is very similar to one another since their diet depends on the scientist^24^. The interesting point is that the rat urine spectra are similar to human urine spectra in this study to a large extent. The raw human urine spectra in this study is believed to be a reference for further studies with the same method. Another contribution of this work is that certain peak positions are successfully assigned to certain contents. Although some regions of the IR spectrum are very complicated, some compounds stand out clearly in the spectrum like creatinine aromatic ring vibration at ~1490 cm^−1^. Further studies and control experiments will be beneficial to correlate the minimum and maximum value of each compound within the normal physiological range and to determine how these amounts show up in the spectrum. These will be tested in a future study to understand the spectral variations still medically accepted to be normal for healthy individuals.

This study presents an artificial urine formulation that mimics human urine and is both economical and practical for use in a variety of studies with different purposes. Our formulation (MP-AU) is compared to human urine and to the most commonly used AU formulations in literature^10,12^ using infrared spectroscopy. Although comparisons are based on infrared spectra, MP-AU is suitable to study with other spectroscopic methods as well since the protocol is formulated using the actual chemical composition of human urine. All AUs show satisfactory physiological properties in terms of pH and SG. However, the FTIR analysis showed differences among the AUs and, based on the results, MP-AU is the closest formulation to human urine. The spectra of BK-AU deviates from human urine at multiple points in the 1500–800 cm^−1^ region. Absorption at 975 cm^−1^ is the most significant deviation from human urine. The authors believe that the spectral mismatch is mainly due to the imbalance among the compounds in terms of concentration. Similarly, CT-AU spectrum also shows differences, particularly in the 1200–900 cm^−1^ region of the spectrum. As mentioned earlier, in the study by Chutipongtanate and Thongboonkerd^12^ normal range of contents were determined using the results of elderly people; thus, CT-AU fails to mimic the urine of healthy young individuals. Although there is no study comparing spectral differences in urine samples from young adults and elderly people, it can be argued that the spectral properties can be affected by the existence of molecules, such as drugs and their metabolites, cytokines or increased different endogen substances seen in various diseases, and the metabolites of some hormones, which can vary with older age.

The spectra of the average human urine and MP-AU show satisfactory agreement in terms of the number of peaks and peak positions. Differences between the two are mainly due to the missing compounds in the MP-AU formulation. In order to mimic human urine with the fewest number of chemicals so that it can be prepared quickly in large volumes, it was decided in this research not to include nucleic acids, amino acids and and proteins (e.g. Tamm Horsfall protein) despite their presence in human urine. The lack of amino acids and proteins did not cause a significant variation that would otherwise be seen in the 1700–1500 cm^−1^ window of the spectrum. On the other hand, the lack of nucleic acids could be a contributor to the variations in the 1200–800 cm^−1^ window. Another factor that might have caused variations between MP-AU and human urine is the age group of the reference values in Mayo Clinic database, in which many of the compounds were determined from a wide age range of people. Some of the spectral differences might also be due to the ethnicity difference between reference people and the volunteers in this study.

Given that there are more than 90 compounds detected by NMR^1^ spectroscopy, for those who wish to adopt a more inclusive approach to human urine and its components, the list of compounds can be further extended. Furthermore, yeast and peptone can be added for bacterial growth studies as also suggested by Brooks and Keevil^10^. For crystal growth studies, the amount of calcium, oxalate or uric acid can be changed^14,45^. For changing the pH of the 24h-mixed final solution, HCl or NaOH can be used, or alternatively, the relative ratio of sodium monobasic and dibasic compounds in the solution can be changed for the desired pH for studying kidney stone formations. The MP-AU presented here is colourless and odourless and, in addition, does not require any special pre-treatment or instrument, hence the advantage and possibility of being prepared for educational purposes. For visual resemblance, adding yellow food dye can also be considered bearing in mind the change in the chemical composition.

## Supplementary information

Supplementary Information.
