# Supplementary material for: A New Artificial Urine Protocol to Better Imitate Human Urine
Source: Sci Rep. 2019 Dec 27;9:20159. doi: 10.1038/s41598-019-56693-4 (PMC6934465; doi:10.1038/s41598-019-56693-4)
Supplement: Supplementary file 1 — Supplementary Information. [file 41598_2019_56693_MOESM1_ESM.docx]

**A New Artificial Urine Protocol to Better Imitate Human Urine**

Neslihan Sarıgül^a*^, Filiz Korkmaz^b^, İlhan Kurultak^c^

^a^ Institute of Nuclear Science, Hacettepe University, 06532 Ankara Turkey

^b^ Atilim University, Faculty of Engineering, Biophysics Laboratory, 06836 Ankara Turkey

^c^ Trakya University, Faculty of Medicine, Department of Nephrology, 22000 Edirne, Turkey

*Corresponding author: Neslihan Sarıgül, PhD, Institute of Nuclear Science, Hacettepe University, 06532 Ankara Turkey. E-mail: [nsarigul@hacettepe.edu.tr](mailto:nsarigul@hacettepe.edu.tr)

**SUPPLEMENTARY MATERIAL**

Table S1. The list of compounds and their concentrations in CT-AU and BK-AU formulations.

|  | **MW (g/mol)** | **Molarity (mM)/**  **CT-AU** | **Molarity (mM)/**  **BK-AU** | **Quantity (g) for 100 ml/**  **CT-AU** | **Quantity (g) for 100 ml/**  **BK-AU** |
| --- | --- | --- | --- | --- | --- |
| CH_4_N_2_O | 60.06 | 200 | 170 | 1.2012 | 1.0210 |
| C_5_H_4_N_4_O_3_ | 168.11 | 1 | 0.4 | 0.0168 | 0.0067 |
| C_4_H_7_N_3_O | 113.12 | 4 | 7 | 0.0452 | 0.0792 |
| Na_3_C_6_H_5_O_7_.2H_2_0 | 294.1 | 5 | 2 | 0.1471 | 0.0588 |
| NaCl | 58.44 | 54 | 90 | 0.3156 | 0.5260 |
| KCl | 74.55 | 30 | - | 0.2237 | - |
| NH_4_Cl | 53.491 | 15 | 25 | 0.0802 | 0.1337 |
| CaCl_2_ | 110.98 | 3 | 2.5 | 0.0333 | 0.0277 |
| MgSO_4_.7H_2_O | 246.48 | 2 | 2 | 0.0493 | 0.0493 |
| NaHCO_3_ | 84.01 | 2 | 25 | 0.0168 | 0.2100 |
| K_2_C_2_O_4_ | 184.24 | 0.1 | - | 0.0018 | - |
| Na_2_SO_4_ | 142.04 | 9 | 10 | 0.1278 | 0.1420 |
| NaH_2_PO_4_.2H_2_O | 156.02 | 3.6 | 7 | 0.0562 | 0.1092 |
| Na_2_HPO_4_.2H_2_O | 177.99 | 0.4 | 7 | 0.0071 | 0.1246 |
| FeSO_4_. 7H_2_O | 278.01 | - | 0.005 | - | 0.0001 |
| Lactic acid | 90.08 | - | 1.1 | - | 0.0099 |
| Pepton* |  | - | - | - | - |
| Yeast extract* |  | - | - | - | - |

*Compound is added for bacterial growth studies in the original paper; it is not used in this study.


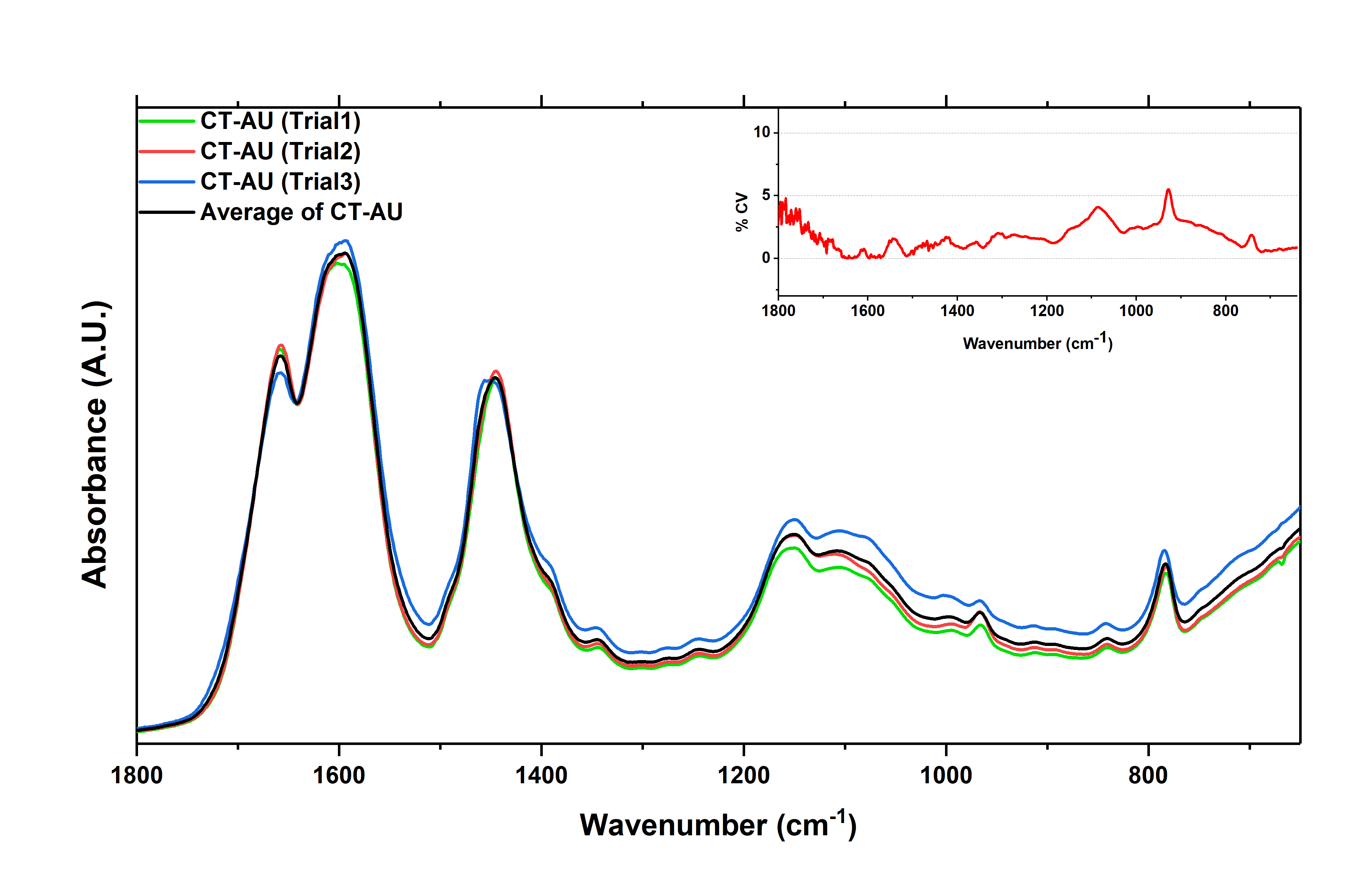

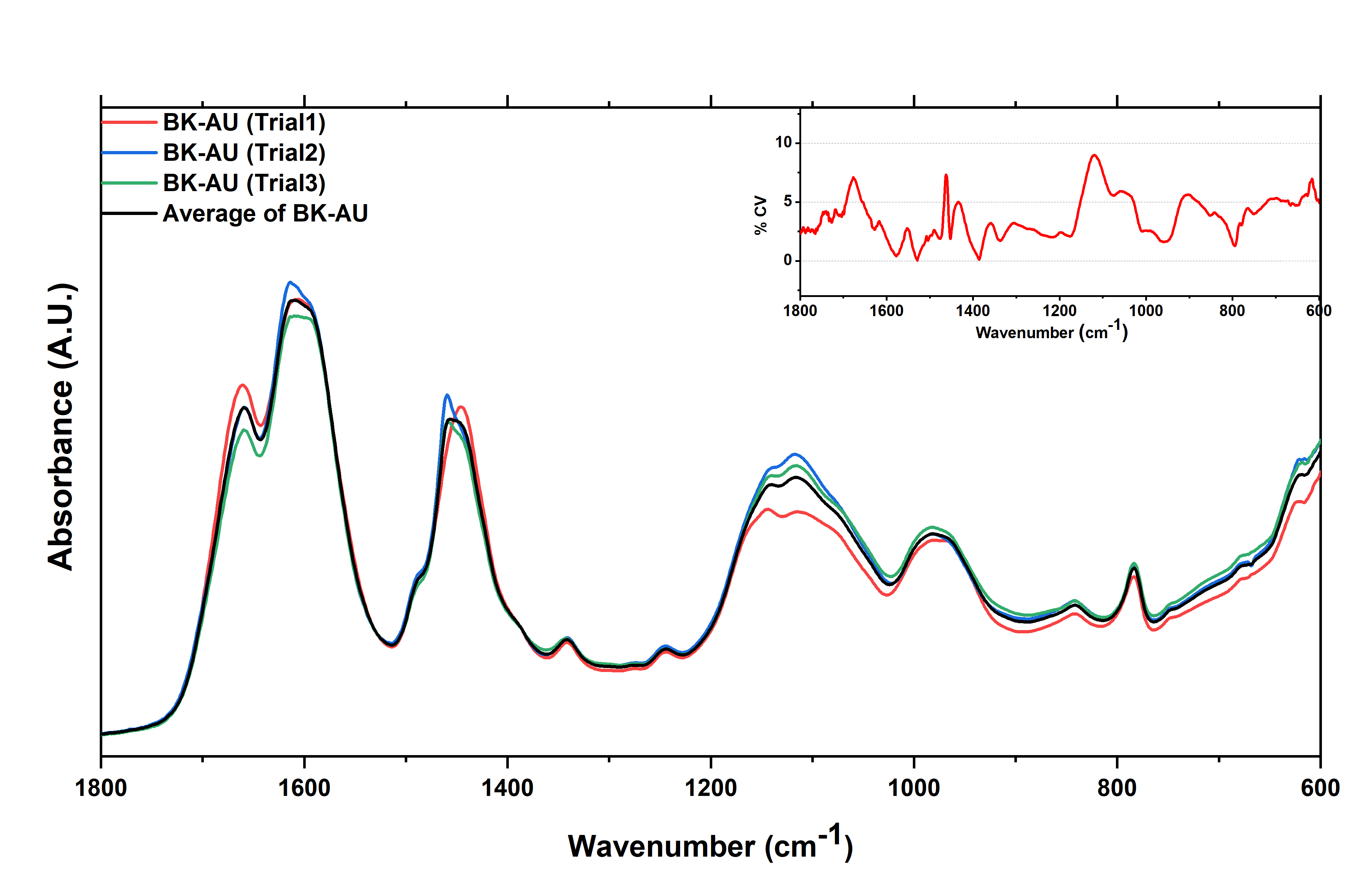

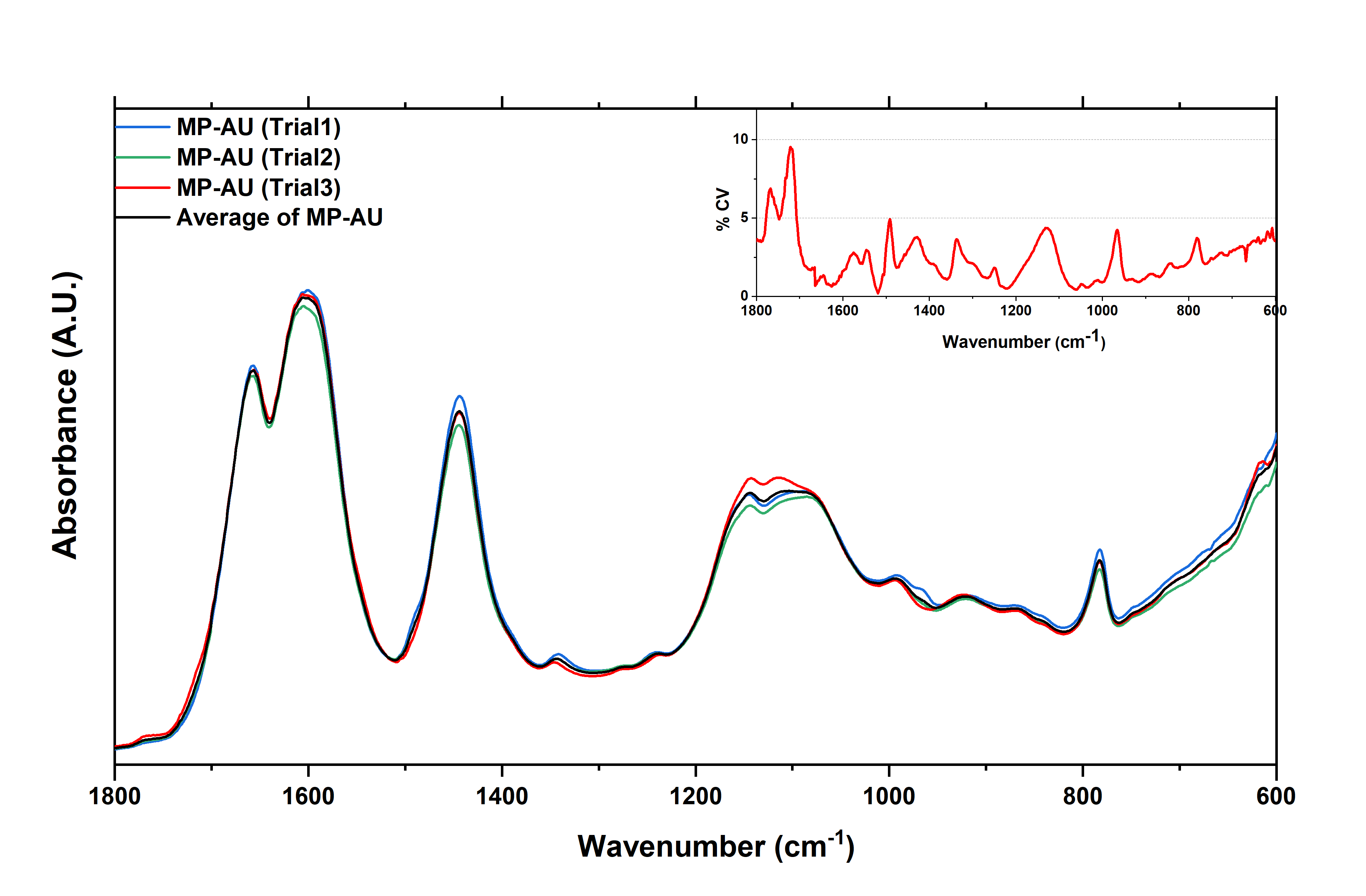


**Figure S1.** Repeatability of all artificial urines are tested by preparing three samples from each and recording their FTIR spectra. Inlet figures show the percentage of coefficient of variation among three measurements for each AU.


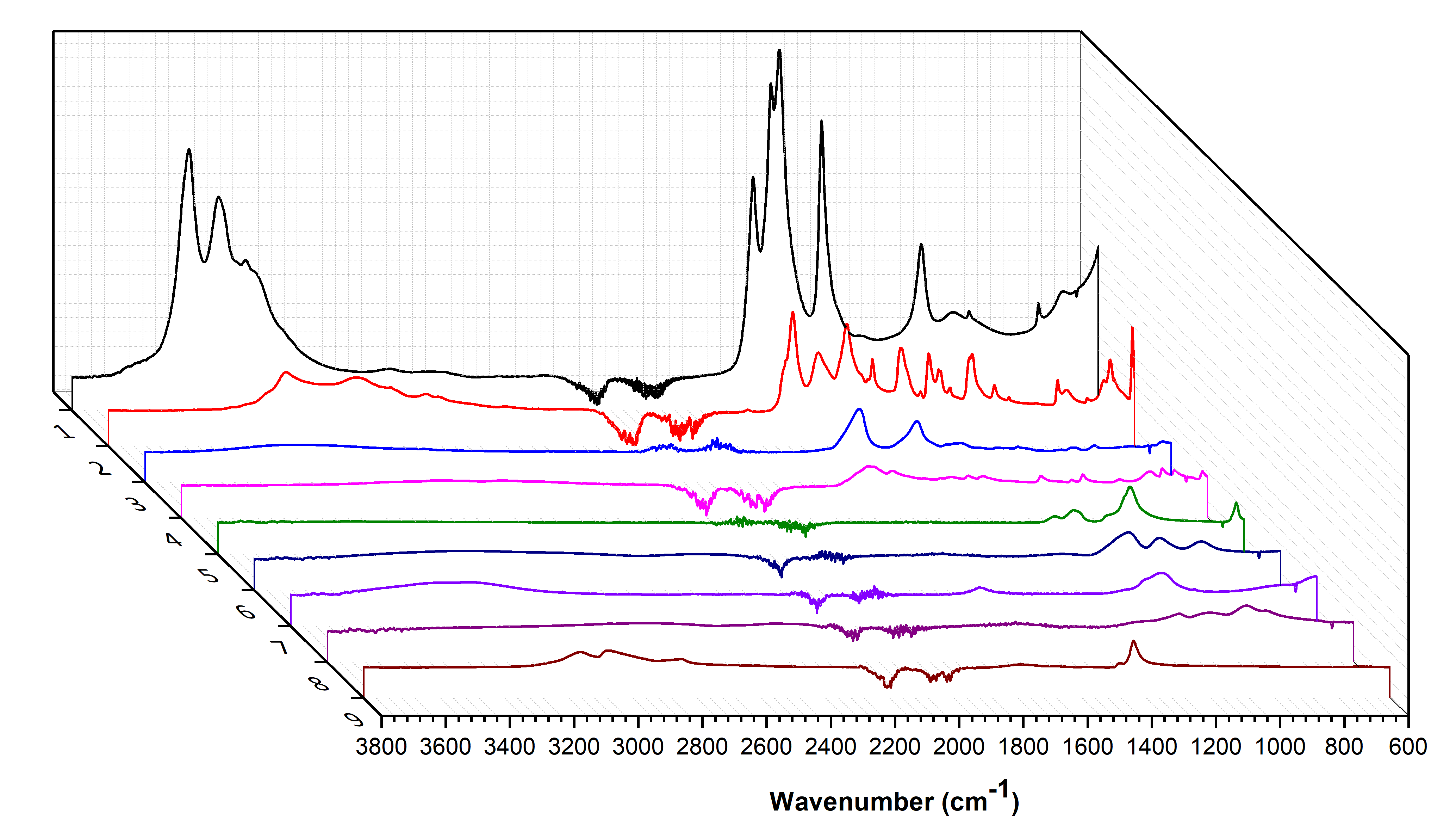


**Figure S2.** Infrared spectra of urea (1), creatinine (2), sodium citrate (3), uric acid (4), sodium sulphate (5), di-sodium hydrogen phosphate (6), magnesium sulphate (7), sodium di-hydrogen phosphate (8), and ammonium cloride (9). The image is constructed to see the relative absorbances. Please refer to Methods section for the details on sample preparation and instrument parameters.

**Table S2.** Position of main infrared absorption bands of compounds (Fig.1 and Fig. S2) used in MP-AU and of bicarbonate (Fig. S3) used in BK-AU and CT-AU.

| **Compound name** | **Main band positions (cm^-1^)** |
| --- | --- |
| Urea (CH_4_N_2_O) | 3434, 3342, 3256, 3223, 2810, 1676, 1621, 1594, 1463, 1331, 1152, 1049, 998, 787, 710 |
| Creatinine (C_4_H_7_N_3_O) | 3245, 3025, 2920,2 807, 2769, 1666, 1587, 1559, 1497, 1432, 1418, 1330, 1268, 1243, 1212, 1205, 1176, 1116, 1107, 1038, 993, 841, 813, 748, 697, 678, 665, 608 |
| Sodium Citrate (Na_3_C_6_H_5_O_7_) | 3319, 2973, 2921, 1573, 1393, 1300, 1256, 1196, 1141, 1110, 1079, 1053, 944, 911, 895, 840, 725, 627 |
| Uric Acid (C_5_H_4_N_4_O_3_) | 3114, 2991, 2918, 2797, 2684, 1657, 1641, 1583, 1485, 1434, 1398, 1347, 1300, 1222, 1120, 1025, 989, 875, 781, 743, 703, 664, 653, 617 |
| Sodium Sulfate (Na_2_SO_4_) | 1190, 1133, 1021, 957, 625 |
| Disodium hydrogen phosphate  (Na_2_HPO_4_) | 3155, 1672, 1365, 1281, 1117, 1082, 980, 851 |
| Magnesium Sulfate(MgSO_4_) | 3327, 3247, 1654, 1201, 1132, 1087, 983, 710 |
| Sodium dihydrogen Phosphate (NaH_2_PO_4)_ | 2772, 1650, 1258, 1145, 1048, 933, 872 |
| Ammonium cloride (NH_4_Cl) | 3123, 3038, 2808, 1752, 1715, 1653, 1546, 1443, 1400, 1260, 1202, 1148, 1017 |
| Potassium oxalate (K_2_C_2_O_4_) | 1589, 1308, 767 |
| Sodium Bicarbonate (NaHCO_3_) | 1731, 1692, 1662, 1621, 1451, 1391, 1301, 1201, 1146, 1046, 1032, 992, 832, 687, 664, 657, 634 |


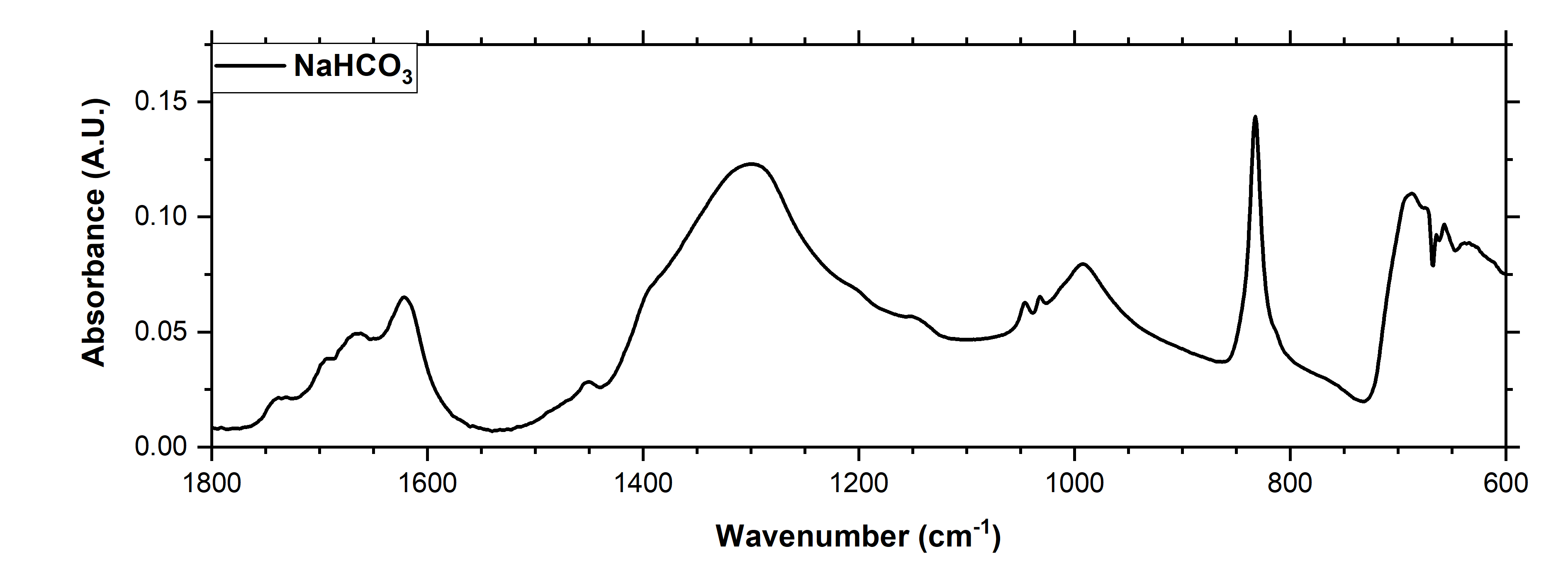


**Figure S3.** Infrared spectrum of 25 mM sodium bicarbonate (NaHCO_3_) used in BK-AU protocol measured with ATR-FTIR.

**Statistical Test Results on FTIR spectra**

Table S3. MP-AU and Healthy average urine comparison

| **Mann-Whitney Test Ranks** | | | |
| --- | --- | --- | --- |
| ***Urines*** | ***N*** | ***Mean Rank*** | ***Sum Rank*** |
| MP-AU | 1246 | 1232 | 1.5E6 |
| Healthy Avg. urine | 1246 | 1261 | 1.6E6 |

| **Mann-Whitney Test Statistic** | | |
| --- | --- | --- |
| ***U*** | ***Z*** | ***Asymp.Prob>\|U\|*** |
| 758238.5 | -1 | 0.3 |
| At the 0.05 level, the two distributions are NOT significantly different. | | |

Table S4. CT-AU and Healthy average urine comparison

| **Mann-Whitney Test Ranks** | | | |
| --- | --- | --- | --- |
| ***Urines*** | ***N*** | ***Mean Rank*** | ***Sum Rank*** |
| CT-AU | 1246 | 1052.1 | 1.3E6 |
| Healthy Avg. urine | 1246 | 1440.9 | 1.8E6 |

| **Mann-Whitney Test Statistic** | | |
| --- | --- | --- |
| ***U*** | ***Z*** | ***Asymp.Prob>\|U\|*** |
| 534054 | -13.5 | 1.9E-41 |
| At the 0.05 level, the two distributions are significantly different. | | |

Table S5. BK-AU and Healthy average urine comparison

| **Mann-Whitney Test Ranks** | | | |
| --- | --- | --- | --- |
| ***Urines*** | ***N*** | ***Mean Rank*** | ***Sum Rank*** |
| BK-AU | 1246 | 1091.3 | 1.4E6 |
| Healthy Avg. urine | 1246 | 1401.7 | 1.7E6 |

| **Mann-Whitney Test Statistic** | | |
| --- | --- | --- |
| U | Z | Asymp.Prob>\|U\| |
| 582885 | -10.8 | 4.9E-27 |
| At the 0.05 level, the two distributions are significantly different. | | |
